# Comparison of CRISPR/Cas9 expression constructs for efficient targeted mutagenesis in rice

**DOI:** 10.1007/s11103-015-0342-x

**Published:** 2015-07-19

**Authors:** Masafumi Mikami, Seiichi Toki, Masaki Endo

**Affiliations:** Graduate School of Nanobioscience, Yokohama City University, 22-2 Seto, Yokohama, Kanagawa 236-0027 Japan; Plant Genome Engineering Research Unit, Agrogenomics Research Center, National Institute of Agrobiological Sciences, 2-1-2 Kannondai, Tsukuba, Ibaraki 305-8602 Japan; Kihara Institute for Biological Research, Yokohama City University, 641-12 Maioka-cho, Yokohama, Kanagawa 244-0813 Japan

**Keywords:** CRISPR/Cas9, Genome editing, Targeted mutagenesis, Rice

## Abstract

The CRISPR/Cas9 system is an efficient tool used for genome editing in a variety of organisms. Despite several recent reports of successful targeted mutagenesis using the CRISPR/Cas9 system in plants, in each case the target gene of interest, the Cas9 expression system and guide-RNA (gRNA) used, and the tissues used for transformation and subsequent mutagenesis differed, hence the reported frequencies of targeted mutagenesis cannot be compared directly. Here, we evaluated mutation frequency in rice using different Cas9 and/or gRNA expression cassettes under standardized experimental conditions. We introduced Cas9 and gRNA expression cassettes separately or sequentially into rice calli, and assessed the frequency of mutagenesis at the same endogenous targeted sequences. Mutation frequencies differed significantly depending on the Cas9 expression cassette used. In addition, a gRNA driven by the OsU6 promoter was superior to one driven by the OsU3 promoter. Using an all-in-one expression vector harboring the best combined Cas9/gRNA expression cassette resulted in a much improved frequency of targeted mutagenesis in rice calli, and bi-allelic mutant plants were produced in the T_0_ generation. The approach presented here could be adapted to optimize the construction of Cas9/gRNA cassettes for genome editing in a variety of plants.

**Electronic supplementary material:**

The online version of this article (doi:10.1007/s11103-015-0342-x) contains supplementary material, which is available to authorized users.

## Introduction

The CRISPR/Cas9 system has emerged rapidly in recent years as a robust technology for targeted mutagenesis in various organisms (Jinek et al. 2012, 2013; Mali et al. 2013; Cong et al. 2013; Cho et al. 2013; Hwang et al. 2013; Jiang et al. 2013a). The system is based on the nuclease activity of Cas9 protein combined with a guide-RNA (gRNA) that binds directly to a 20-nt sequence on the target DNA. The precise location of CRISPR/Cas9-mediated DNA cleavage is determined jointly by this 20-nt sequence in the gRNA and the requisite binding region—a NGG motif located immediately after the 20-nt target DNA—which in Cas9 is known as the protospacer adjacent motif (PAM) (Jinek et al. 2012).

Several recent reports describe successful CRISPR/Cas9-mediated targeted mutagenesis in plants (Nekrasov et al. 2013; Shan et al. 2013; Li et al. 2013; Belhaj et al. 2013 for review; Baltes and Voytas 2014 for review). In the field of plant genome engineering, direct delivery of RNA to plant nuclei is difficult. Constructs expressing Cas9 and/or gRNA are usually delivered into plant cells via a DNA vector, thus Cas9 regulatory elements (e.g. promoter and terminator), codon usage for the Cas9 gene and regulatory elements for gRNA transcription must be optimized. Thus, selection of appropriate expression constructs for both gRNA and Cas9 is important. Recently, Johnson et al. (2014), adapting their previously reported assay for cleavage-dependent luciferase gene correction in *Nicotiana benthamiana* leaves (Johnson et al. 2013), conducted a comparative study of different Cas9 constructs using *N. benthamiana* and found significant differences in cleavage efficiency between Cas9 variants. In their study, human and *Arabidopsis thaliana* codon-optimized Cas9 genes showed higher cleavage efficiency compared to Cas9 variants codon-optimized for dicotyledonous plants.

There have been several reports of successful CRISPR/Cas9-mediated targeted mutagenesis in rice (Baltes and Voytas 2014 for review); however, the target genes, Cas9 and gRNA expression systems, tissues used for transformation and mutagenesis, and the method of evaluation of mutation frequency differ in each report (see Belhaj et al. 2013 for review). In some cases, mutation efficiency was evaluated by transient assay using PEG-mediated transformation of protoplasts (Shan et al. 2013; Xie and Yang 2013). In other reports, Cas9 and gRNA expression constructs were transformed into calli by Agrobacterium-mediated transformation, and the mutation frequency was inferred from the ratio of regenerated plants containing mutations (Feng et al. 2013; Mao et al. 2013; Miao et al. 2013; Xie and Yang 2013; Jiang et al. 2013b; Xu et al. 2014; Zhang et al. 2014; Zhou et al. 2014; Endo et al. 2015). Because the expression level of the transgene varies among independent transgenic callus clones due to differences in copy number and positioning of the transgene, the ratio of mutated plants differs in transgenic lines even if the same constructs are transformed. Furthermore, any prolonged callus culture period increases the proportion of mutated cells (Mikami et al. 2015). Hence, direct comparison of the results described in different papers is impossible.

In this study, in order to select the optimal combination of Cas9 and gRNA constructs for targeted mutagenesis in rice, we introduced Cas9 and gRNA expression cassettes into rice calli either separately or sequentially via *Agrobacterium*-mediated transformation, and evaluated the frequency of targeted mutagenesis in the resulting calli using the same target sequences and uniform criteria.

## Materials and methods

### Construction of Cas9 expression vectors

Our original Cas9 expression vectors, pZH_MMomegaCas9, pZH_MMCas9 and pZH_AtomegaCas9, were constructed as follows: (1) the Cas9 ORF was codon-optimized for rice or *Arabidopsis*, respectively, by Fasmac (Kanagawa, Japan). (2) Connected sequences of the translational enhancer sequences of the Cauliflower mosaic virus (CaMV) omega sequence or rice ADH 5′ UTR, codon-optimized Cas9, and the pea rbcS3A (pea3A) terminator sequence were synthesized flanked by *Xba*I and *Pac*I sites. (3) These synthesized fragments (MMomegaCas9::Tpea3A, OsADH5′UTR::MMCas9::Tpea3A and AtomegaCas9::Tpea3A) were cloned into pE(L3-L2) downstream of the double CaMV 35S promoter (2xP35S) using *Xba*I and *Pac*I sites. (4) 2xP35S::MMomegaCas9::Tpea3A, 2xP35S::OsADH5′UTR::MMCas9::Tpea3A and 2xP35S::AtomegaCas9::Tpea3A fragments were digested with *Asc*I and *Pac*I and cloned into pZH with a hygromycin resistance cassette [2xP35S::HPT::TnosT35S.] (Kuroda et al. 2010).

pZH_hCas9 was constructed as follows: (1) pK7WGF2::hCas9 (Nekrasov et al. 2013) was obtained from Addgene (www.addgene.org). (2) pK7WGF2::hCas9 was excised with *Spe*I and *Kpn*I and, the EGFP::hCas9::T35S fragment was cloned into pZH downstream of the 2xP35S promoter. pZH_modified-hspCas9 was constructed as follows: (1) the fragment [2x35S:: OsADH5′UTR::hspCas9 (Feng et al. 2013)::TNos] was synthesized by Fasmac. (2) This synthesized fragment was cloned into pZH using *Asc*I and *Pac*I sites.

pZH_FFCas9 was constructed as follows: (1) the Pcubi::Cas9::Tpea3A fragment in pDe-CAS9 (Fauser et al. 2014) was cloned into pZH using *Pvu*II and *Avr*II sites.

### Construction of gRNA expression vectors

Target sequences used in this study are shown in Online Resource 1. The gRNA expression vectors of gYSA (pZK_gRNA) were constructed as follows: (1) OsU3 or OsU6 promoter sequences, 20-nt target sequence, gRNA scaffold and poly T were synthesized by Fasmac flanked by *Asc*I and *Pac*I sites. (2) This synthesized fragment was cloned into pZK with a kanamycin resistance cassette [2xP35S::NPTII::T35S] using *Asc*I and *Pac*I sites.

### Construction of gRNA/Cas9 all-in-one vectors

The pZH_OsU6gRNA_MMCas9 vector was constructed as follows: (1) the synthesized OsU3::gYSA, 2xP35S::OsADH5′UTR::MMCas9::Tpea3A, and OsAct1 3′UTR::P35S::HPT::Thsp17.3 fragments were cloned into pPZP202 via an In-fusion cloning reaction (Takara, Shiga, Japan). (2) Double-stranded target sequences were made by annealing the paired single oligonucleotides shown in Online Resource 2: the gRNA cloning vector, pU6gRNA-oligo and pU3gRNA-oligo has two *Bbs*I and *Bsa*I sites between the OsU6 or OsU3 promoter and the gRNA scaffold sequence. These vectors were linearized using *Bbs*I and *Bsa*I, respectively, and the 20-nt annealed oligo-nucleotides were ligated into these restriction enzyme recognition sites (similar to Fauser et al. 2014) (3) OsU3-gYSA in pZH_OsU3gYSA_MMCas9 was replaced by synthetic gRNA expression constructs using *Asc*I and *Pac*I sites.

### Transformation of Cas9 and gRNA expression constructs

*Agrobacterium*-mediated transformation of rice (*Oryza sativa* L. cv. Nipponbare) using scutellum-derived calli was performed as described previously (Toki 1997; Toki et al. 2006). One-month-cultured rice calli were infected by *Agrobacterium* carrying the pZH_Cas9 vector in a first transformation. After 3 days of co-cultivation, infected calli were transferred to fresh callus-induction medium (CIM) (Toki 1997) containing 50 mg/L hygromycin B (Wako Pure Chemicals, Osaka, Japan) and 25 mg/L meropenem (Wako) to remove *Agrobacterium.* Hygromycin-resistant calli were selected over 6 weeks. Proliferating calli were then transferred to fresh CIM without meropenem and cultured for 1 week. Next, these calli were infected by *Agrobacterium* carrying the pZK_gRNA vector in a second round of transformation. After 3 days of co-cultivation, infected calli were transferred to fresh CIM containing 35 mg/L geneticin 418 (Nakarai, Kyoto, Japan) and 25 mg/L meropenem. After 4 weeks of selection, transgenic calli of pZH_Cas9 and pZK_gRNA were used for analysis of mutation frequency. In the case of the all-in-one Cas9/gRNA expression constructs (pZH_OsU6gRNA_MMCas9), 3-week-old cultured rice scutellum-derived calli were used for transformation, and mutation frequency was analyzed 4 weeks after transformation.

### CAPS analysis

DNA was extracted from calli or regenerated plants, and target loci amplified using the primers listed in Online Resource 2. PCR products were subjected to restriction enzyme digestion (Online Resource 1) and analyzed by agarose gel electrophoresis.

### Sequencing analysis

PCR products used for CAPS analysis were cloned into pCR-BluntII-TOPO (Invitrogen, San Diego, CA) and subjected to sequence analysis using an ABI3130 sequencer (Applied Biosystems, Foster City, CA).

## Results

### Construction and expression of Cas9 and gRNAs

**Fig. 1:**
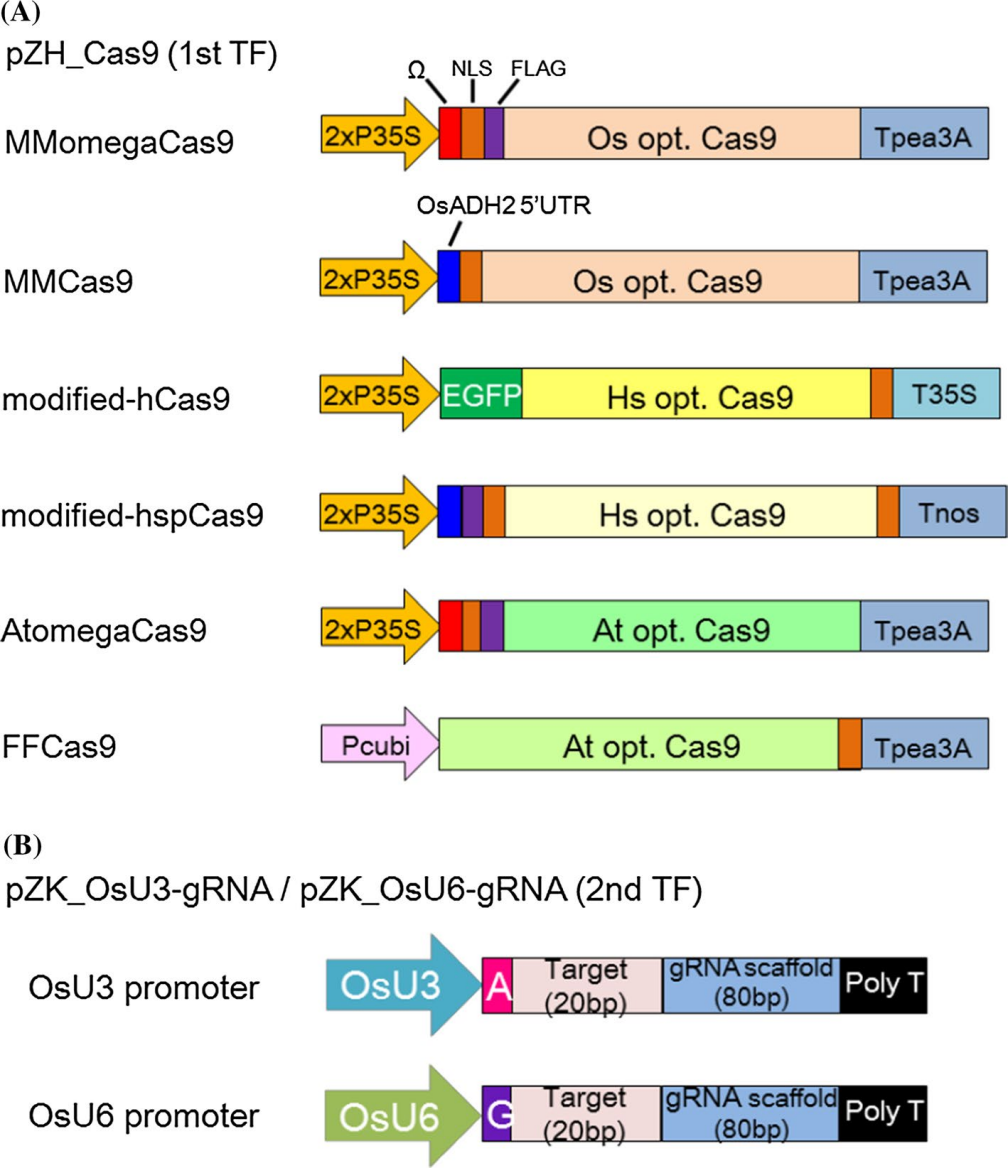
Expression constructs for Cas9 and gRNA. **a** Cas9 expression constructs. Os opt., Hs opt., and At opt. Cas9 indicate Cas9 codon-optimized for rice, human, and *Arabidopsis*, respectively. *Different colors* indicate different codon usage. pZH_Cas9 vectors, each with one of these Cas9 expression cassettes, together with an HPT expression construct, were transformed into rice calli in a first round of transformation (1st TF). **b** gRNA expression constructs with a 20-bp target sequence. A and G refer to the first transcription point of the OsU3 and OsU6 promoter, respectively. These gRNA expression constructs were introduced into the vector pZK to construct pZK_OsU3-gRNA and pZK_OsU6-gRNA for use in a second transformation (2nd TF)

The Cas9 expression constructs used in this experiment are shown in Fig. 1a. The Cas9 coding sequence was either newly synthesized for this study or provided by Addgene (www.addgene.org). Vectors MMomegaCas9 and MMCas9 were codon-modified for rice. The translational enhancer sequence of the CaMV omega sequence (Mitsuhara et al. 1996) was used in MMomegaCas9 and that of the 5′-UTR from the *O. sativa* alcohol dehydrogenease 2 gene (*OsADH2* 5′-UTR: Sugio et al. 2009) was used in MMCas9. A modified-hCas9 vector was constructed from pK7WGF2::hCas9 (Nekrasov et al. 2013), which uses Cas9 codon-modified for humans. Our modified-hspCas9 vector was also codon-modified for humans and was derived from hspCas9 (Feng et al. 2013). The AtomegaCas9 vector was codon-modified for *Arabidopsis* and also includes the CaMV omega sequence. FFCas9 vector was derived from pDe_CAS9 (Fauser et al. 2014) and codon-modified for *Arabidopsis*. In all vectors except modified-pDe-Cas9, the Cas9 coding region was driven by the CaMV 2x35S promoter and cloned into the pZH vector (Kuroda et al. 2010) harboring a hygromycin resistance gene as a selection marker. In the FFCas9 vector, Cas9 was driven by the PcUbi4-2 promoter (Fauser et al. 2014).

The gRNA was expressed under the control of either the *OsU3* promoter from the rice U3 small nuclear RNA (snRNA) gene (Wang et al. 2008) or the *OsU6* promoter from the rice U6-2 snRNA gene (Feng et al. 2013) (Fig. 1b). Both gRNA expression cassettes were cloned into pZK (Kuroda et al. 2010) with a kanamycin resistance gene as a selection marker (Fig. 1b).

**Fig. 2:**
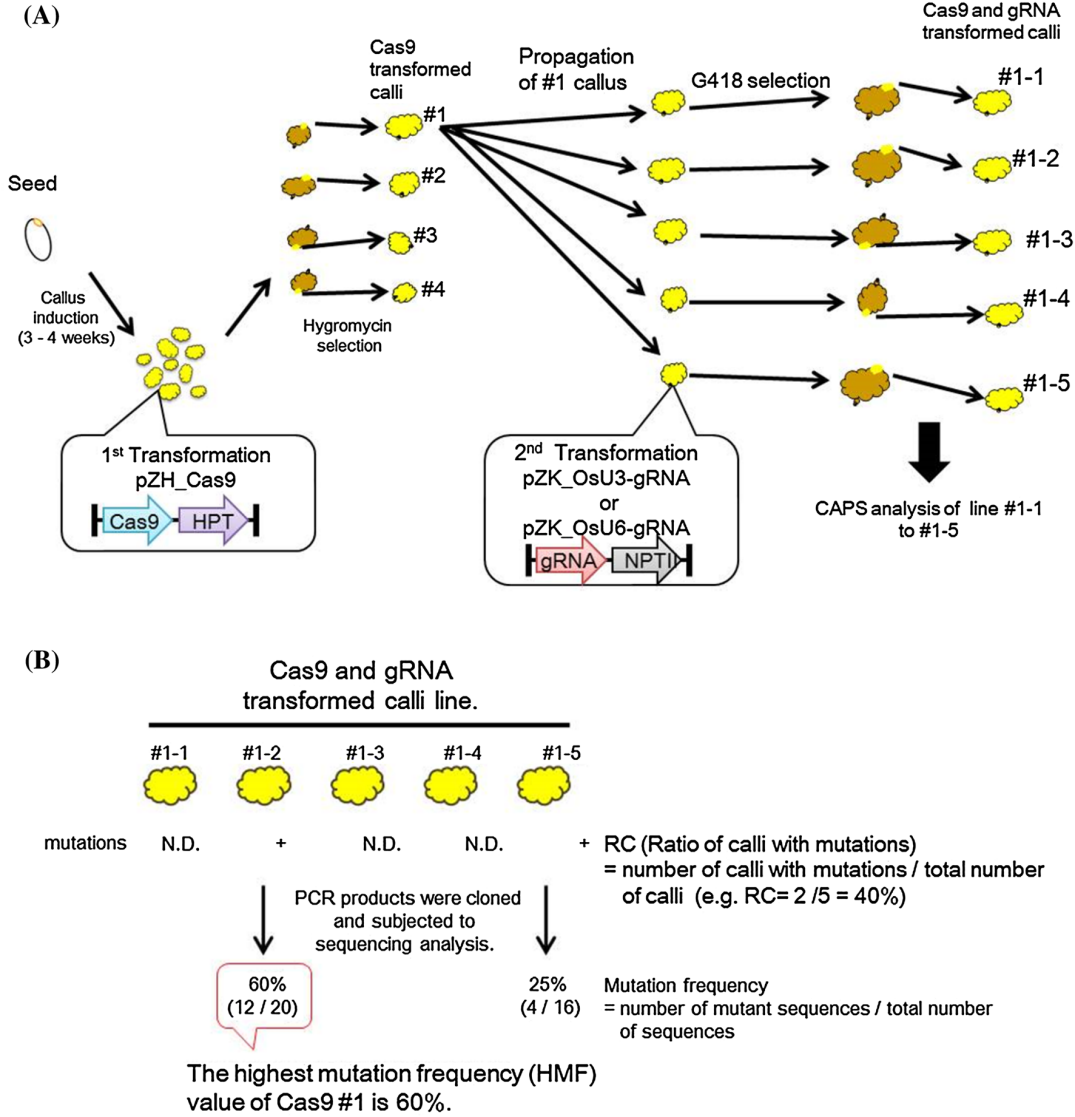
Schematic representation of the CRISPR/Cas9-mediated target mutagenesis employed in this study. **a** Process of transformation and rules of numbering. **b** Evaluation of mutation frequency

As shown in Fig. 2a, the Cas9 expression vector (pZH_Cas9) was introduced into 1-month-old scutellum-derived rice calli via *Agrobacterium*-mediated transformation. Cas9-transformed rice calli were selected on hygromycin sulfate for 3 weeks then propagated further for 1 month. The gRNA expression vector (pZK_gRNA) was then introduced into the Cas9-transformed calli in a second round of transformation. After another month of selection against geneticin, total genomic DNA was extracted and subjected to cleaved amplified polymorphic sequences (CAPS) analysis to reveal the presence of mutations at the targeted sequence. Since the CRISPR/Cas9 cleavage site was designed to contain a restriction enzyme recognition sequence on the target gene, mutated sequences became susceptible to restriction enzyme digestion.

We evaluated the frequency of targeted mutagenesis in two ways. The first method utilised the CAPS assay and simply scored the number of calli with mutated CRISPR/Cas9 relative to the total number of calli transformed with Cas9 and gRNA cassettes. We define this criteria as the “ratio of calli with mutations” (RC; see Fig. 2b). Our second evaluation criterion was to measure mutation frequency in the most effectively mutated callus, termed “highest mutation frequency” (HMF; see Fig. 2b). PCR products of clonally propagated calli were cloned, and the ratio of mutated to non-mutated sequence evaluated by DNA sequencing.

### Selection of Cas9 expression cassettes optimal for rice target mutagenesis

Rice calli were transformed with different pZH_Cas9 vectors (Fig. 1a), and independent transgenic lines (e.g. #1–#4) were obtained for each Cas9 construct (see Fig. 2a). Then, in a second round of transformation, pZK_OsU3-gRNA was introduced into one of these independent transgenic lines (e.g. #1) and further independent Cas9/gRNA doubly transformed transgenic calli were obtained (e.g. #1-1 to #1-5; see Fig. 2a).

**Fig. 3:**
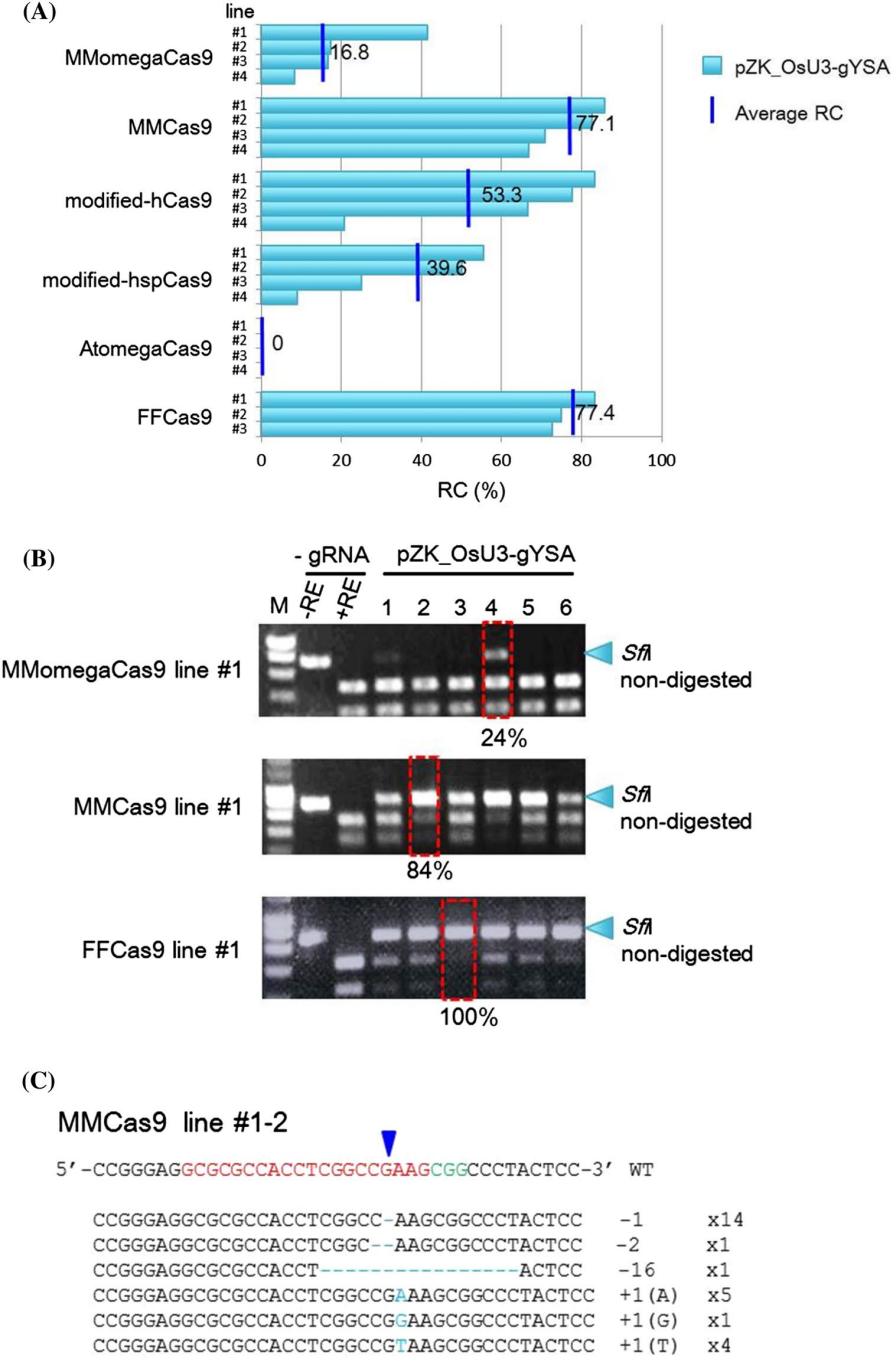
Comparison of Cas9 expression constructs. **a** Ratio of calli with mutations (RC) in different Cas9 expression constructs. DNAs extracted from pZH_Cas9 and pZK_OsU3-gYSA transformed calli were subjected to PCR and subsequent *Sfi*I restriction enzyme digestion. *Blue bars* show the average RC in independent Cas9 transgenic *lines*, #1–#4 (or #3). **b** CAPS analysis of the gYSA locus in MMomegaCas9, MMCas9 and FFCas9 line #1. *M* Marker; −*RE* PCR product without restriction enzyme reaction; +*RE* *Sfi*I-digested PCR product; -*gRNA* pZH_Cas9 transformed calli without pZK_OsU3-gYSA transformation; the HMF score was calculated from the PCR products indicated within *red rectangles*. **c** Mutations detected by sequence analysis of DNA extracted from MMCas9 #1–2 calli. The wild type sequence is shown at the *top* with the PAM sequence in *green*, and the 20 nt target sequence in *red*. The *blue arrowhead* indicates the expected cleavage site. *Dashes* deleted bases. The net changes in length are shown to the *right* of each sequence (+ insertion; − deletion). The number of clones representing each mutant allele is shown in *brackets*

In the same way, pZK_OsU3-gRNA was transformed to Cas9 lines #2–#4. The *young seedling albino* (YSA; Feng et al. 2013) gene was selected as the target of modification in this experiment (Table S1), since bi-allelic mutants show a typical albino phenotype. To detect mutations in the *YSA* gene, we conducted CAPS analysis using DNA extracted from Cas9- and gRNA-transformed calli (e.g. #1-1 to #1-6, see Fig. 2a). The targeted mutation was detected with all Cas9 expression cassettes except AtomegaCas9 (Fig. 3a). The average RC scores in the two most highly mutated lines (MMCas9 and FFCas9) were 77.1 and 77.4 %, respectively (Fig. 3a). The HMF value of MMCas9 was 84 % in line #1–2 and that of FFCas9 was 100 % in line #1–3 (Fig. 3b). The various mutations detected in line #1–2 transformed with MMCas9 are shown in Fig. 3c. Since a variety of mutations, including a 1-nt deletion (−1), a large deletion (−16) and 1 nt insertions (+1) of A, T or G were observed with different rates of emergence, we concluded that proliferation of mutated cells and de novo mutations could occur in parallel in small clonally propagated calli expressing Cas9 and gRNA constructs. The average RC in MMomegaCas9 was 16.8 % and the corresponding HMF score was 24 % in line #1–4; both these values were lower than those of the two most highly mutated Cas9 expression cassettes: MMCas9 and FFCas9 (Fig. 3a, b). These findings suggested that the results of evaluation of mutation frequency using the RC and HMF criteria are somewhat correlated. Because the RC and HMF scores were relatively high in MMCas9 and FFCas9-transformed calli, these two constructs were deemed optimal for rice targeted mutagenesis. In fact, when we analyzed 34 plants regenerated from a single transgenic callus (MMCas9 #1-1; Fig. 3b) with a mutation frequency of 56 %, 5 plants were mono-allelic mutants and 23 plants were bi-allelic mutants (Online Resource 3).

### Comparison of OsU3 and OsU6 promoters for gRNA expression

**Fig. 4:**
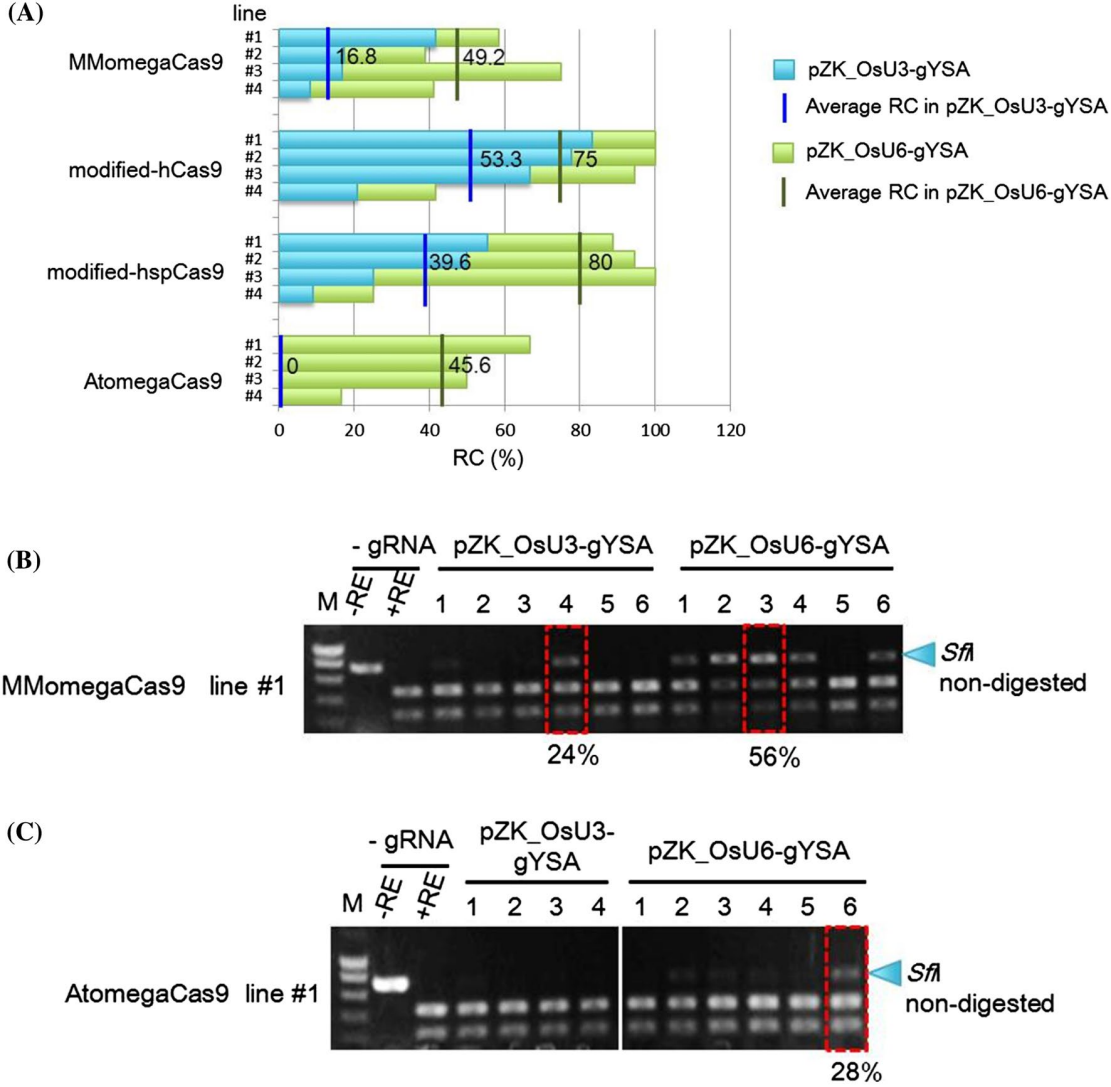
Comparison of gRNA expression cassettes. **a** RCs in pZH_Cas9 and pZK_OsU3-gYSA or pZK_OsU6-gYSA transformed calli. pZK_OsU3-gYSA and pZK_OsU6-gYSA were transformed into the same clonally propagated transgenic callus of pZH_Cas9. DNAs extracted from double transformed calli of pZH_Cas9 and pZK_OsU3-gYSA or pZK_OsU6-gYSA were subjected to PCR and subsequent *Sfi*I restriction enzyme digestion. *Blue bars* show the average RC in independent Cas9 transgenic *lines* #1–#4 (or #3) transformed with pZK_OsU3-gYSA. *Green bars* show the average RC in independent Cas9 transgenic *lines* #1–#4 (or #3) transformed with pZK_OsU6-gYSA. **b**, **c** CAPS analysis of the gYSA locus in MMomegaCas9, AtomegaCas9 line #1 transformed with pZK_OsU3-gYSA or pZK_OsU6-gYSA. HMFs in pZH_Cas9 and pZK_OsU3-gYSA or pZK_OsU6-gYSA double transformed calli are indicated by *red rectangles*

Next, we compared the effect on the frequency of targeted mutagenesis of using the OsU3 or OsU6 promoter for gRNA expression. The gRNA expression constructs pZK_OsU3-gYSA or pZK_OsU6-gYSA, which targeted the *YSA* gene, were transformed into the same clonally propagated transgenic callus of different Cas9 expression cassettes. When mutation frequency was evaluated with RC criteria, the OsU6 promoter performed much better than the OsU3 promoter (Fig. 4a). The OsU6 promoter was also predominant when HMF criteria were used for evaluation, namely, the HMF value of MMomegaCas9 line #1 increased from 24 to 56 % upon changing the gRNA construct from pZK_OsU3-gYSA to pZK_OsU6-gYSA (Fig. 4b). Even AtomegaCas9-transformed callus, which did not contain mutated cells when pZK_OsU3-gYSA was used for gRNA expression, gave a high mutation score when pZK_OsU6-gYSA was used; the average RC was 45.6 % (21 out of 46) and HMF value was 28 % (Fig. 4c). These results indicate that the mutation frequency was increased significantly when using OsU6 rather than the OsU3 promoter for gRNA expression.

### Targeted mutagenesis using an all-in-one Cas9/gRNA vector

**Fig. 5:**
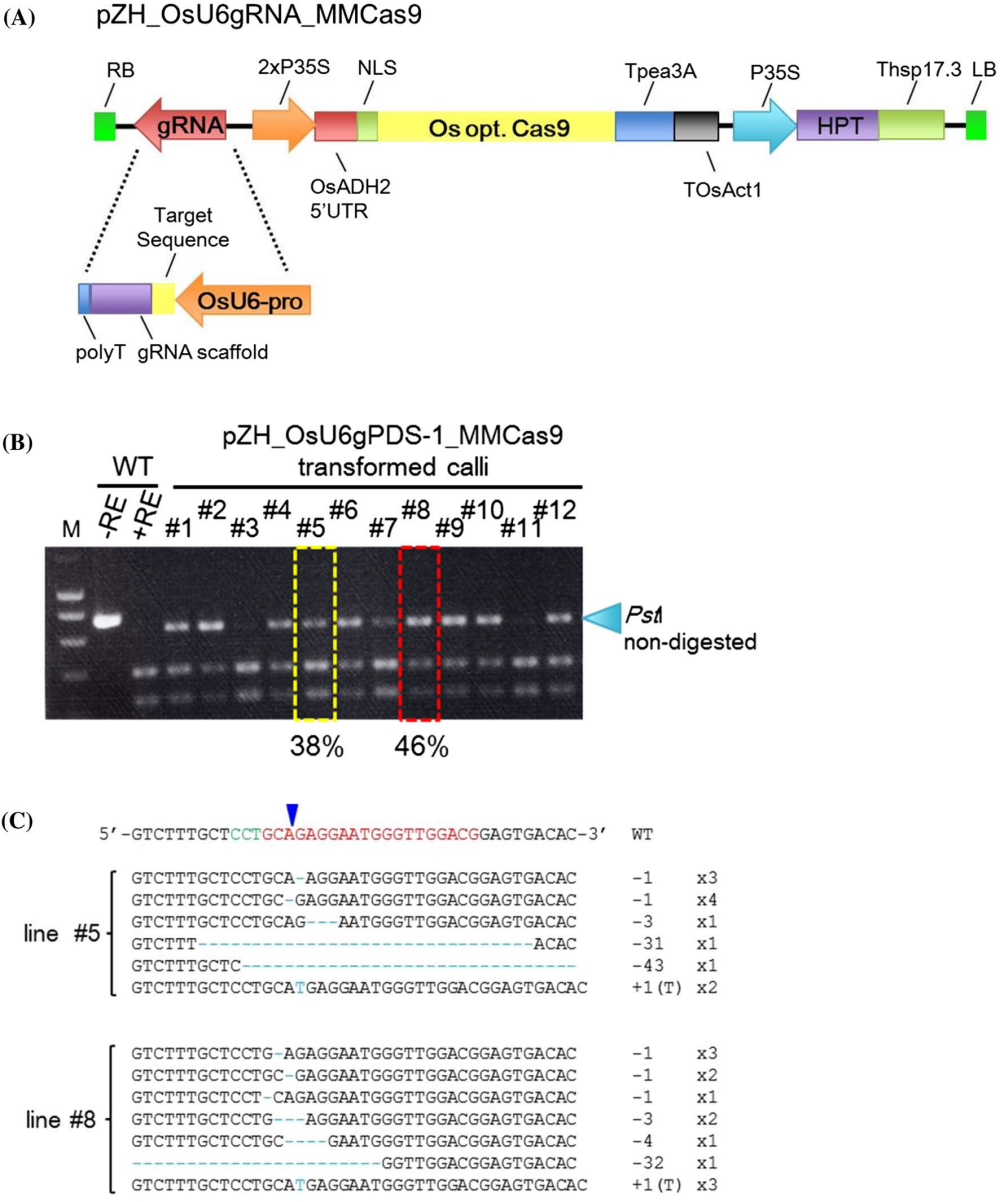
Targeted mutagenesis using pZH_OsU6gRNA_MMCas9 vector. **a** Construction of pZH_OsU6gRNA_MMCas9 vector. **b** CAPS analysis of the gPDS-1 target locus. DNAs extracted from independent pZH_OsU6gPDS-1_MMCas9 transformed calli were subjected to PCR and subsequent *Pst*I restriction enzyme digestion. *WT* non-transgenic callus lines. The HMF score is shown under the *red rectangle*. Mutation frequency in the other line #5 is shown under the *yellow rectangle*. **c** Mutations detected in lines #5 and #8

The results above indicated that efficient targeted mutagenesis can be achieved when MMCas9 or FFCas9 is expressed together with OsU6-gRNA. Since delivery of the Cas9 expression cassette and the OsU6-gRNA expression cassette separately by sequential *Agrobacterium*-mediated transformation is laborious, we constructed an all-in-one vector to express MMCas9, OsU6-gRNA and a hygromycin resistance gene (pZH_OsU6gRNA_MMCas9; see Fig. 5a).

**Fig. 6:**
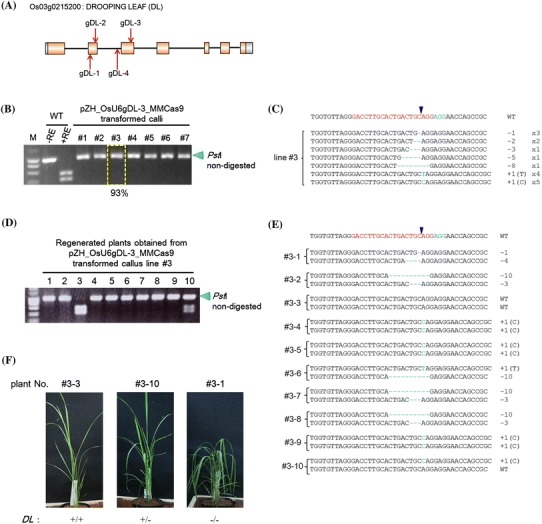
Targeted mutagenesis in the rice *DL* gene. **a** Target sites of CRISPR/Cas9-mediated target mutagenesis in the rice *DL* gene. gDL-1, 2, 3, 4 are located on exons 2 and 3 and intron 2, respectively. **b** CAPS analysis of the gDL-3 target locus in pZH_OsU6gDL-3_MMCas9-transformed calli. In the 7 transgenic lines analyzed (#1–7), few cleaved PCR products were detected. Mutation frequency in callus line #3 estimated by sequencing of the cloned PCR product is 93 %. **c** Mutation variations detected in #3 by sequencing analysis. **d** CAPS analysis of the gDL-3 target locus in regenerated plants obtained from callus line #3. **e** Variation of mutation detected in regenerated plants. **f** Phenotypes of *dl* mutant plants. The bi-allelic mutant plant #3-1 showed the drooping leaves phenotype

The effectiveness of this all-in-one vector was evaluated using the *phytoene desaturase* (PDS) and *drooping leaf* (DL; Yamaguchi et al. 2004; Ohmori et al. 2011) genes as targets. In one-month-old cultured calli transformed with this all-in-one vector targeting PDS (gPDS-1, Online Resource 1), the RC was 90.4 % (19 out of 21) and the HMF value was 46 % (Fig. 5b). Details of mutations detected in lines #5 and #8 are shown in Fig. 5c. A 1-nt deletion (−1) and a 1 nt insertion (+1) occurred in calli of both lines #5 and #8. In addition, the ratio of mutated plants regenerated from callus line #5 was 42.1 % (8 out of 19), and one plant possessing a bi-allelic mutation in the PDS gene showed an albino phenotype. In a further experiment, 4 loci in the *DL* gene were selected as target sequences for pZH_OsU6gRNA_MMCas9-mediated mutagenesis (Fig. 6a; Table 1, Online Resource 4). In the case of gDL-3, the RC was 100 % (11 out of 11), and the HMF value around 93 % (Fig. 6b). Various types of mutation were detected in callus line #3 (Fig. 6c). Of ten regenerated plants obtained from line #3 callus, 8 were bi-allelic mutants, one was a mono-allelic mutant and one a non-mutated wild-type plant (Fig. 6d, e). The five bi-allelic mutant plants showed a drooping leaves phenotype (Fig. 6f; e.g. #3–1); however, the three bi-allelic mutant plants with 10-nt and 3-nt deletions (#3–2, #3–7, #3–8; Fig. 6e) did not exhibit this phenotype (Table S3). Mutations in other target sites in the DL gene (gDL-1, gDL-2 or gDL-4) were also induced with high efficiency in transgenic calli and various kinds of mutations were detected in clonally propagated calli (Online Resource 4). Many bi-allelic mutant plants were obtained (Online Resource 5); bi-allelic mutants of gDL-1 and gDL-2 harboring an insertion or deletion in the exon showed the drooping leaves phenotype while bi-allelic mutant plants of gDL-4, which targeted the intron, did not (Online Resource 6).

**Table 1:** Summary of mutation frequency in pZH_OsU6gRNA_MMCas9 transformed calli

Target gene	gRNA name	Mutation frequency		Were bi-allelic mutant plants regenerated?
		RC (%)	HMF (%)	
PDS	gPDS-1^a^	19/20 (95)	46	Yes
	gPDS-2	1/12 (8.3)	3	No
	gPDS-3	11/11 (100)	93	Yes
	gPDS-4	11/11 (100)	87	Yes
DL	gDL-1	11/11 (100)	68	Yes
	gDL-2	11/11 (100)	94	Yes
	gDL-3	11/11 (100)	93	Yes
	gDL-4	11/11 (100)	76	Yes
LigIV	gLigIV-1	20/20 (100)	62	Yes
	gLigIV-2	21/21 (100)	90	Yes
	gLigIV-3	21/21 (100)	96	Yes
	gLigIV-4	21/21 (100)	88	Yes
ALS	gALS-1	4/9 (44)	5	No^b^
	gALS-2	8/8 (100)	73	No^b^
	gALS-3	9/9 (100)	69	No^b^
	gALS-4	8/9 (88)	26	No^b^

^a^gPDS-1 refers to the OsPDS-SP2 target locus (Shan et al. 2013) on the PDS gene

^b^Because the knockout cells of ALS gene did not survive, bi-allelic mutant plants were not regenerated. But mono-allelic mutant plants in gALS-2, -3, -4 were regenerated except in the case of gALS-1

Finally, we succeeded in inducing mutations in other target genes with high efficiency using a similar all-in-one vector, and obtained bi-allelic mutants (Table 1). Our results indicate that the MMCas9 and FFCas9 vector will be very useful for targeted mutagenesis in rice.

## Discussion

Since the direct delivery of mRNA encoding Cas9 and gRNA is still difficult in plants, optimization of Cas9/gRNA expression constructs remains an important goal for plant genome engineering. Although many successful reports of targeted mutagenesis in rice have now been published, ranking of vectors used in these studies has been impossible to date because target genes, and methods of evaluating mutation efficiency are different in each report. Here, we evaluated different Cas9 and gRNA expression constructs under the same experimental conditions. We compared mutation efficiency using 6 different Cas9 expression constructs and 2 gRNA expression constructs, introducing Cas9 and gRNA constructs separately into rice calli via *Agrobacterium*-mediated transformation; we found that mutation efficiency varied greatly depending on the Cas9 and gRNA expression constructs used. MMCas9 and FFCas9 were selected as the best Cas9 expression constructs for rice targeted mutagenesis (Fig. 3a). Regarding gRNA expression constructs, mutation frequency was higher when the OsU6 promoter was used than with the OsU3 promoter (Fig. 4a). Armed with these results, an all-in-one vector harboring MMCas9 and OsU6-gRNA expression constructs (pZH_OsU6gRNA_MMCas9) was established, and high frequency mutagenesis in multiple target genes using these vectors was confirmed (Table 1). Furthermore, many bi-allelic mutant plants were regenerated from the highly frequent mutagenized calli obtained using this all-in-one vector.

MMomegaCas9 and MMCas9 showed differences in mutation frequency (Fig. 3a). These constructs vary in the translational enhancer used (omega for MMomegaCas9, *OsADH2* 5′UTR for MMCas9) and in whether the FLAG sequence is present (MMomegaCas9) or absent (MMCas9) (Fig. 1a). In other experiments, we have shown that Cas9 expression level and mutation frequency are positively correlated (Mikami et al. 2015). Furthermore, the *OsADH2* 5′UTR is reported to be more effective than omega in promoting high levels of translation in rice (Sugio et al. 2009). Our results here indicate that the amount of Cas9 protein expressed in MMCas9-transformed calli was higher than that in MMomegaCas9. In addition, different mutation frequencies in MMomegaCas9 and AtomegaCas9 seemed to stem from the codon usage of Cas9 (Fig. 1a). In this regard, Li et al. (2013) showed different Cas9 protein expression levels and different mutation frequency in *Arabidopsis* protoplasts transformed with a Cas9 gene optimized differently for plants and mammals. We thus expected that codon usage of Cas9 affects stability and/or splicing pattern of mRNA and consequently the amount of functional Cas9 protein in plant cells.

Needless to say, the Cas9 constructs compared in this study represent only a small proportion of Cas9 genes used in rice. Because promoter, terminator, translational enhancer, Cas9 codon usage, and the number and location of nuclear localization signals all affect the amount of functional Cas9 and eventually affect the mutation frequency, improvement of Cas9 expression constructs has the potential to increase mutation frequency. A detailed analysis of each component affecting the expression level of Cas9 and mutation efficiency will be the subject of future work.

In addition to the Cas9 sequences used, target sequences also have a significant impact on mutation frequency. Selection of promising target sequences by in vitro DNA cleavage assay may help the success of in vivo CRISPR/Cas9-mediated targeted mutagenesis. In addition, we found that prolonged culture of Cas9- and gRNA-transformed calli enhanced mutation frequency (Mikami et al. 2015). The use of an appropriate Cas9/gRNA expression construct and optimization of the culture period might be useful in developing the efficient targeted mutagenesis required to address the needs of plant science and molecular breeding in plants.

## Electronic supplementary material

Supplementary material 1 (PPTX 398 kb)

## Abbreviations

CAPS: Cleaved amplified polymorphic sequences
Cas9: CRISPR-associated endonuclease 9
CIM: Callus induction medium
CRISPR: Clustered regularly interspaced short palindromic repeat
DL: Drooping leaf
gRNA: Guide RNA
HMF: Highest mutation frequency
NLS: Nuclear localization signal
nt: Nucleotide
PAM: Protospacer adjacent motif
PDS: Phytoene desaturase
RT-PCR: Reverse transcriptase-polymerase chain reaction
RC: Ratio of calli with mutations
snRNA: Small nuclear RNA
YSA: Young seedling albino
