# Supplementary material for: Comparison of CRISPR/Cas9 expression constructs for efficient targeted mutagenesis in rice
Source: Plant Mol Biol. 2015 Jul 19;88(6):561–72. doi: 10.1007/s11103-015-0342-x (PMC4523696; doi:10.1007/s11103-015-0342-x)
Supplement: Supplementary file 1 — Supplementary material 1 (PPTX 398 kb) [file 11103_2015_342_MOESM1_ESM.pptx]

## Slide 1
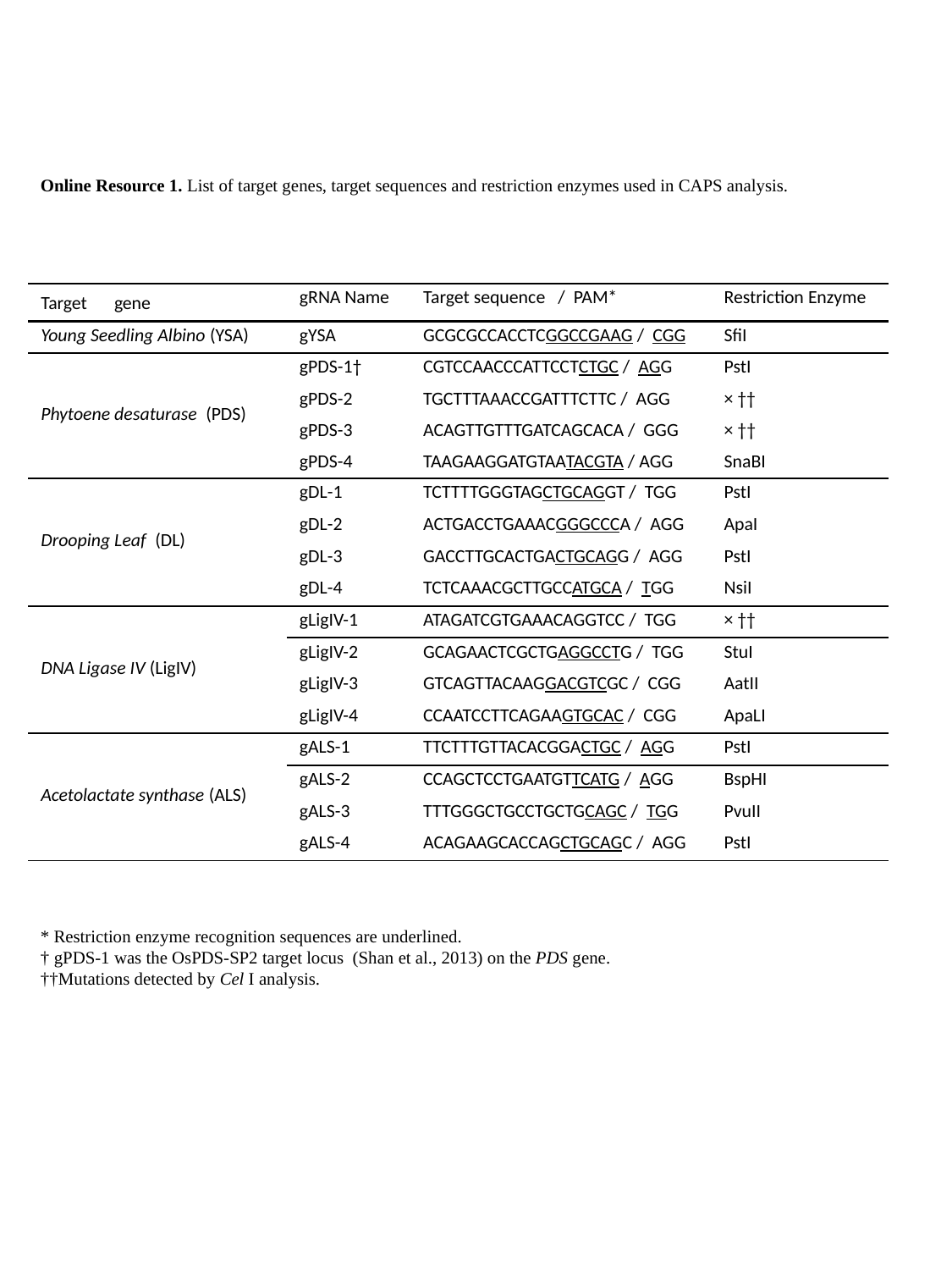

Online Resource 1. List of target genes, target sequences and restriction enzymes used in CAPS analysis.
| Target　gene | gRNA Name | Target sequence / PAM\* | Restriction Enzyme |
| --- | --- | --- | --- |
| Young Seedling Albino (YSA) | gYSA | GCGCGCCACCTCGGCCGAAG / CGG | SfiI |
| Phytoene desaturase (PDS) | gPDS-1† | CGTCCAACCCATTCCTCTGC / AGG | PstI |
| | gPDS-2 | TGCTTTAAACCGATTTCTTC / AGG | × †† |
| | gPDS-3 | ACAGTTGTTTGATCAGCACA / GGG | × †† |
| | gPDS-4 | TAAGAAGGATGTAATACGTA / AGG | SnaBI |
| Drooping Leaf (DL) | gDL-1 | TCTTTTGGGTAGCTGCAGGT / TGG | PstI |
| | gDL-2 | ACTGACCTGAAACGGGCCCA / AGG | ApaI |
| | gDL-3 | GACCTTGCACTGACTGCAGG / AGG | PstI |
| | gDL-4 | TCTCAAACGCTTGCCATGCA / TGG | NsiI |
| DNA Ligase IV (LigIV) | gLigIV-1 | ATAGATCGTGAAACAGGTCC / TGG | × †† |
| | gLigIV-2 | GCAGAACTCGCTGAGGCCTG / TGG | StuI |
| | gLigIV-3 | GTCAGTTACAAGGACGTCGC / CGG | AatII |
| | gLigIV-4 | CCAATCCTTCAGAAGTGCAC / CGG | ApaLI |
| Acetolactate synthase (ALS) | gALS-1 | TTCTTTGTTACACGGACTGC / AGG | PstI |
| | gALS-2 | CCAGCTCCTGAATGTTCATG / AGG | BspHI |
| | gALS-3 | TTTGGGCTGCCTGCTGCAGC / TGG | PvuII |
| | gALS-4 | ACAGAAGCACCAGCTGCAGC / AGG | PstI |
| | | | |
* Restriction enzyme recognition sequences are underlined.
† gPDS-1 was the OsPDS-SP2 target locus  (Shan et al., 2013) on the PDS gene.
††Mutations detected by Cel I analysis.

## Slide 2
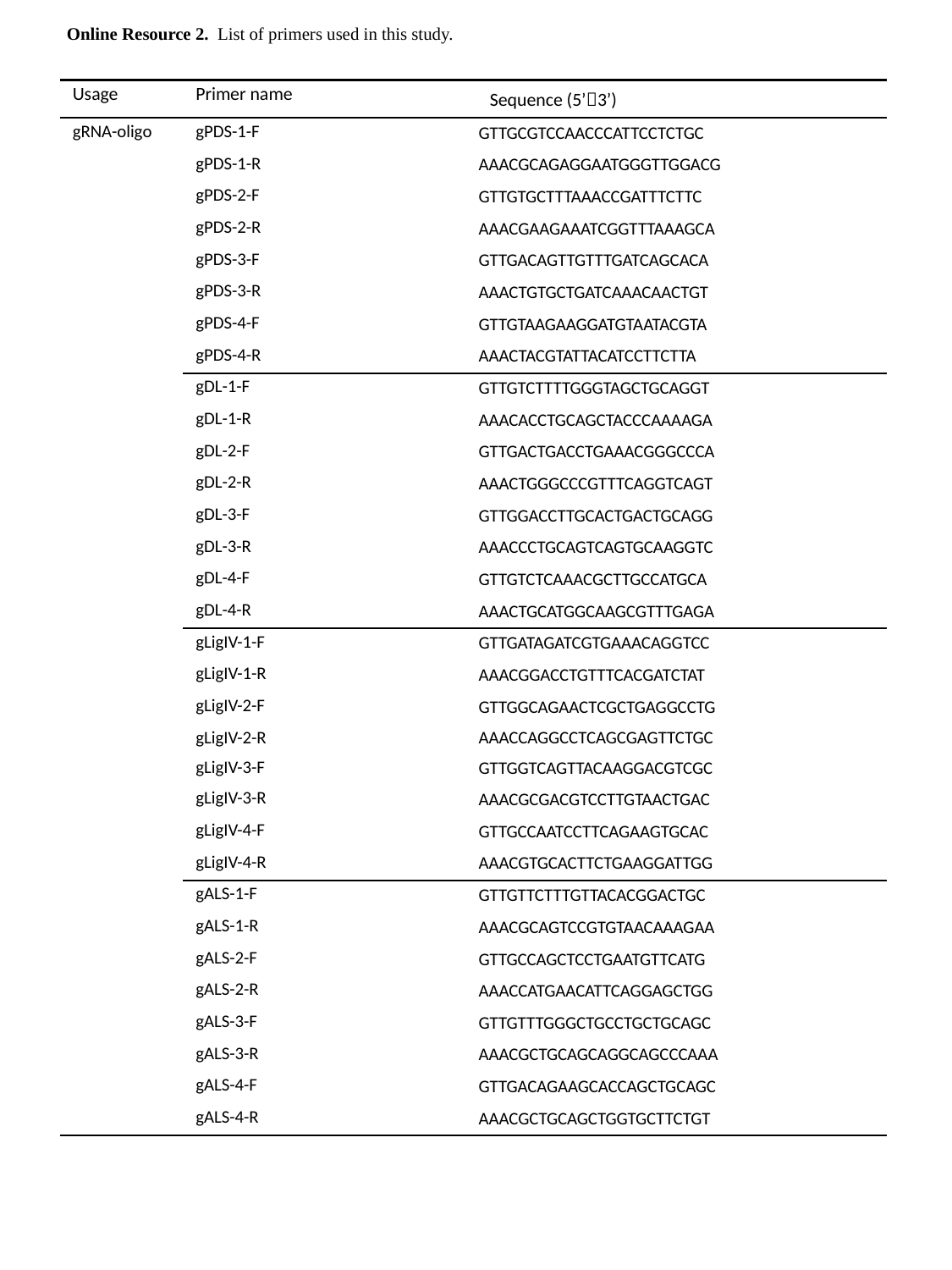

Online Resource 2. List of primers used in this study.
| Usage | Primer name | Sequence (5’3’) |
| --- | --- | --- |
| gRNA-oligo | gPDS-1-F | GTTGCGTCCAACCCATTCCTCTGC |
| | gPDS-1-R | AAACGCAGAGGAATGGGTTGGACG |
| | gPDS-2-F | GTTGTGCTTTAAACCGATTTCTTC |
| | gPDS-2-R | AAACGAAGAAATCGGTTTAAAGCA |
| | gPDS-3-F | GTTGACAGTTGTTTGATCAGCACA |
| | gPDS-3-R | AAACTGTGCTGATCAAACAACTGT |
| | gPDS-4-F | GTTGTAAGAAGGATGTAATACGTA |
| | gPDS-4-R | AAACTACGTATTACATCCTTCTTA |
| | gDL-1-F | GTTGTCTTTTGGGTAGCTGCAGGT |
| | gDL-1-R | AAACACCTGCAGCTACCCAAAAGA |
| | gDL-2-F | GTTGACTGACCTGAAACGGGCCCA |
| | gDL-2-R | AAACTGGGCCCGTTTCAGGTCAGT |
| | gDL-3-F | GTTGGACCTTGCACTGACTGCAGG |
| | gDL-3-R | AAACCCTGCAGTCAGTGCAAGGTC |
| | gDL-4-F | GTTGTCTCAAACGCTTGCCATGCA |
| | gDL-4-R | AAACTGCATGGCAAGCGTTTGAGA |
| | gLigIV-1-F | GTTGATAGATCGTGAAACAGGTCC |
| | gLigIV-1-R | AAACGGACCTGTTTCACGATCTAT |
| | gLigIV-2-F | GTTGGCAGAACTCGCTGAGGCCTG |
| | gLigIV-2-R | AAACCAGGCCTCAGCGAGTTCTGC |
| | gLigIV-3-F | GTTGGTCAGTTACAAGGACGTCGC |
| | gLigIV-3-R | AAACGCGACGTCCTTGTAACTGAC |
| | gLigIV-4-F | GTTGCCAATCCTTCAGAAGTGCAC |
| | gLigIV-4-R | AAACGTGCACTTCTGAAGGATTGG |
| | gALS-1-F | GTTGTTCTTTGTTACACGGACTGC |
| | gALS-1-R | AAACGCAGTCCGTGTAACAAAGAA |
| | gALS-2-F | GTTGCCAGCTCCTGAATGTTCATG |
| | gALS-2-R | AAACCATGAACATTCAGGAGCTGG |
| | gALS-3-F | GTTGTTTGGGCTGCCTGCTGCAGC |
| | gALS-3-R | AAACGCTGCAGCAGGCAGCCCAAA |
| | gALS-4-F | GTTGACAGAAGCACCAGCTGCAGC |
| | gALS-4-R | AAACGCTGCAGCTGGTGCTTCTGT |

## Slide 3
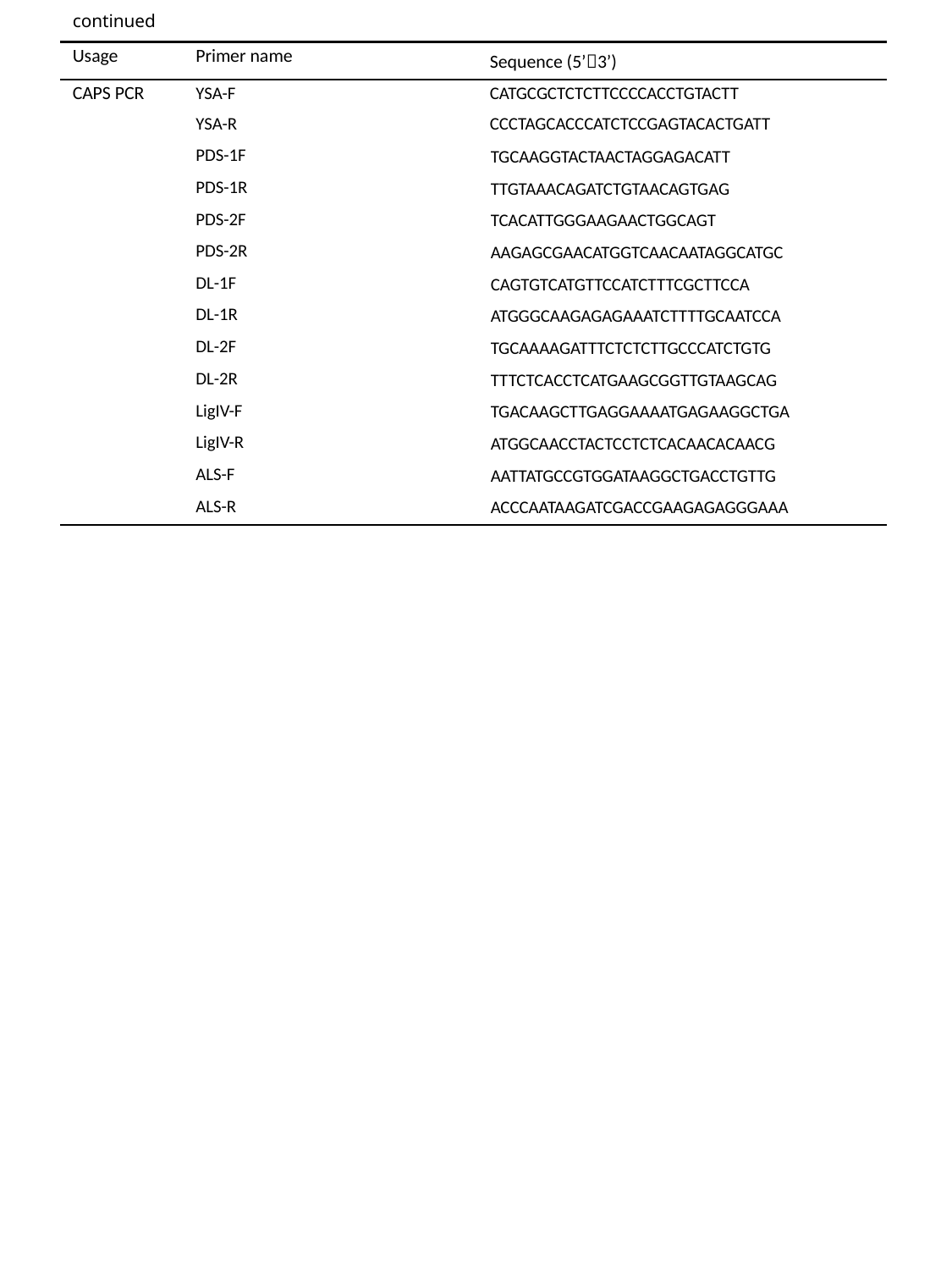

| continued | |
| --- | --- |
| Usage | Primer name | Sequence (5’3’) |
| --- | --- | --- |
| CAPS PCR | YSA-F | CATGCGCTCTCTTCCCCACCTGTACTT |
| | YSA-R | CCCTAGCACCCATCTCCGAGTACACTGATT |
| | PDS-1F | TGCAAGGTACTAACTAGGAGACATT |
| | PDS-1R | TTGTAAACAGATCTGTAACAGTGAG |
| | PDS-2F | TCACATTGGGAAGAACTGGCAGT |
| | PDS-2R | AAGAGCGAACATGGTCAACAATAGGCATGC |
| | DL-1F | CAGTGTCATGTTCCATCTTTCGCTTCCA |
| | DL-1R | ATGGGCAAGAGAGAAATCTTTTGCAATCCA |
| | DL-2F | TGCAAAAGATTTCTCTCTTGCCCATCTGTG |
| | DL-2R | TTTCTCACCTCATGAAGCGGTTGTAAGCAG |
| | LigIV-F | TGACAAGCTTGAGGAAAATGAGAAGGCTGA |
| | LigIV-R | ATGGCAACCTACTCCTCTCACAACACAACG |
| | ALS-F | AATTATGCCGTGGATAAGGCTGACCTGTTG |
| | ALS-R | ACCCAATAAGATCGACCGAAGAGAGGGAAA |

## Slide 4
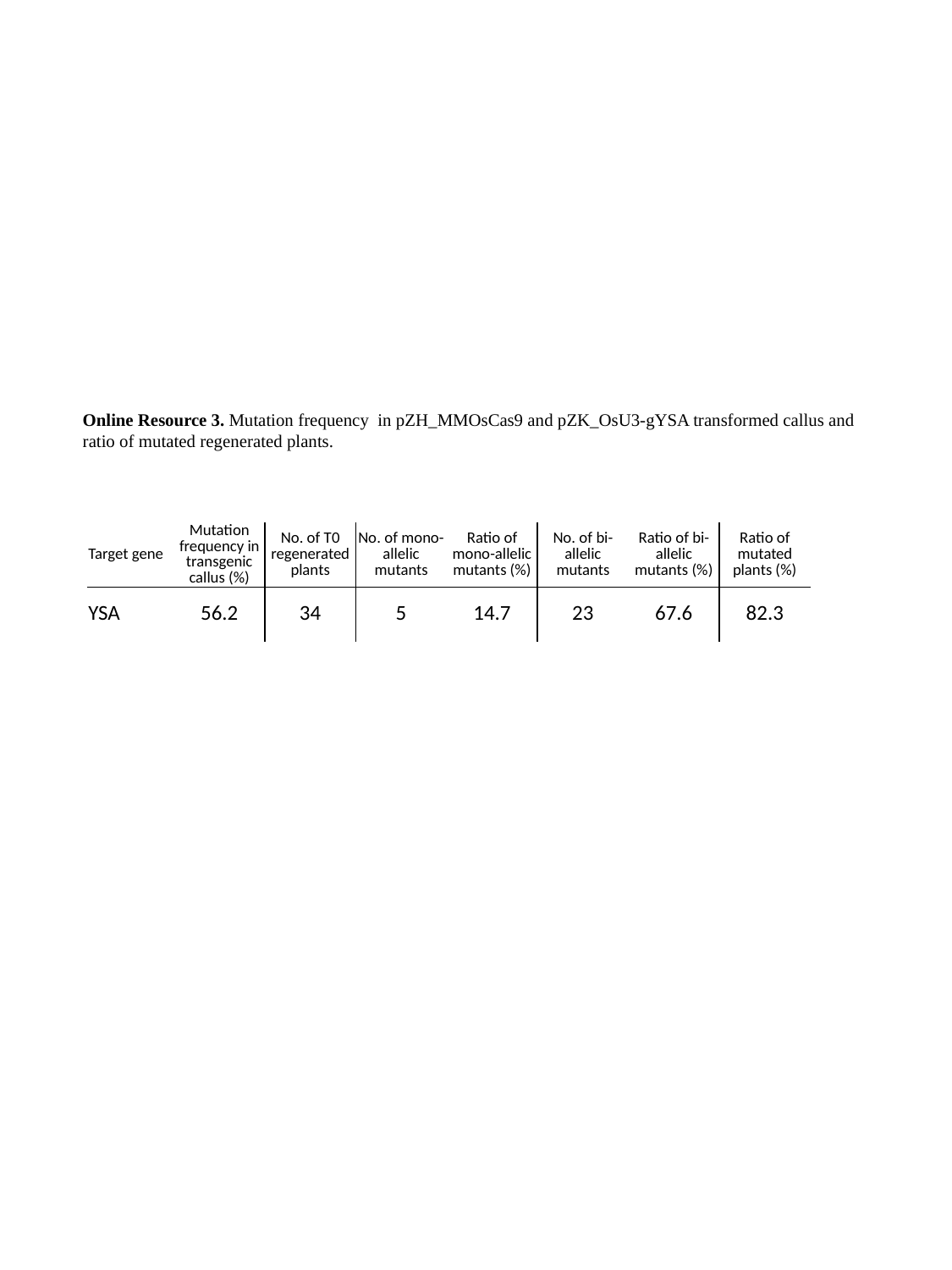

Online Resource 3. Mutation frequency in pZH_MMOsCas9 and pZK_OsU3-gYSA transformed callus and ratio of mutated regenerated plants.
| Target gene | Mutation frequency in transgenic callus (%) | No. of T0 regenerated plants | No. of mono-allelic mutants | Ratio of mono-allelic mutants (%) | No. of bi-allelic mutants | Ratio of bi-allelic mutants (%) | Ratio of mutated plants (%) |
| --- | --- | --- | --- | --- | --- | --- | --- |
| YSA | 56.2 | 34 | 5 | 14.7 | 23 | 67.6 | 82.3 |

## Slide 5
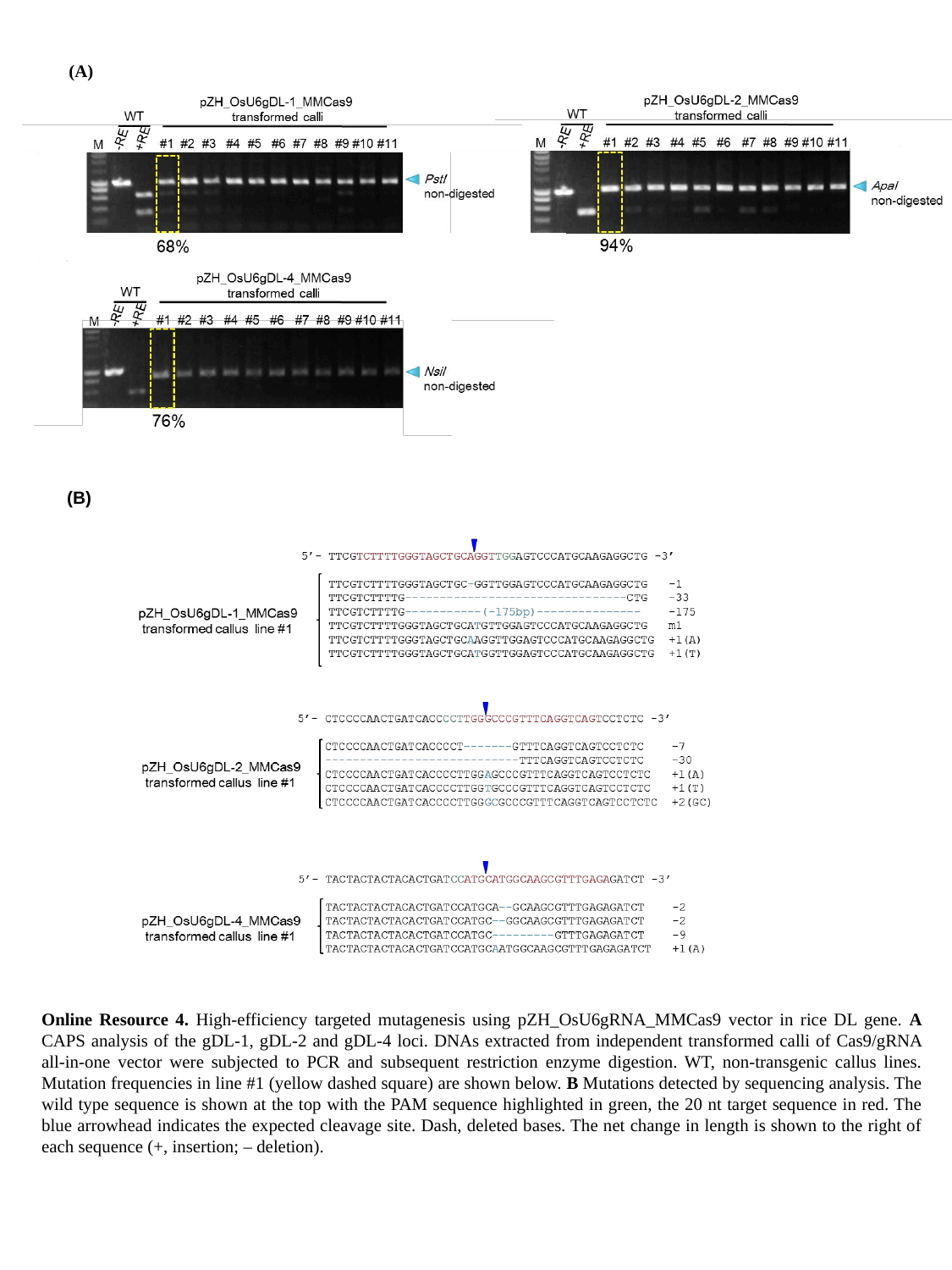

(A)
(B)
Online Resource 4. High-efficiency targeted mutagenesis using pZH_OsU6gRNA_MMCas9 vector in rice DL gene. A CAPS analysis of the gDL-1, gDL-2 and gDL-4 loci. DNAs extracted from independent transformed calli of Cas9/gRNA all-in-one vector were subjected to PCR and subsequent restriction enzyme digestion. WT, non-transgenic callus lines. Mutation frequencies in line #1 (yellow dashed square) are shown below. B Mutations detected by sequencing analysis. The wild type sequence is shown at the top with the PAM sequence highlighted in green, the 20 nt target sequence in red. The blue arrowhead indicates the expected cleavage site. Dash, deleted bases. The net change in length is shown to the right of each sequence (+, insertion; – deletion).

## Slide 6
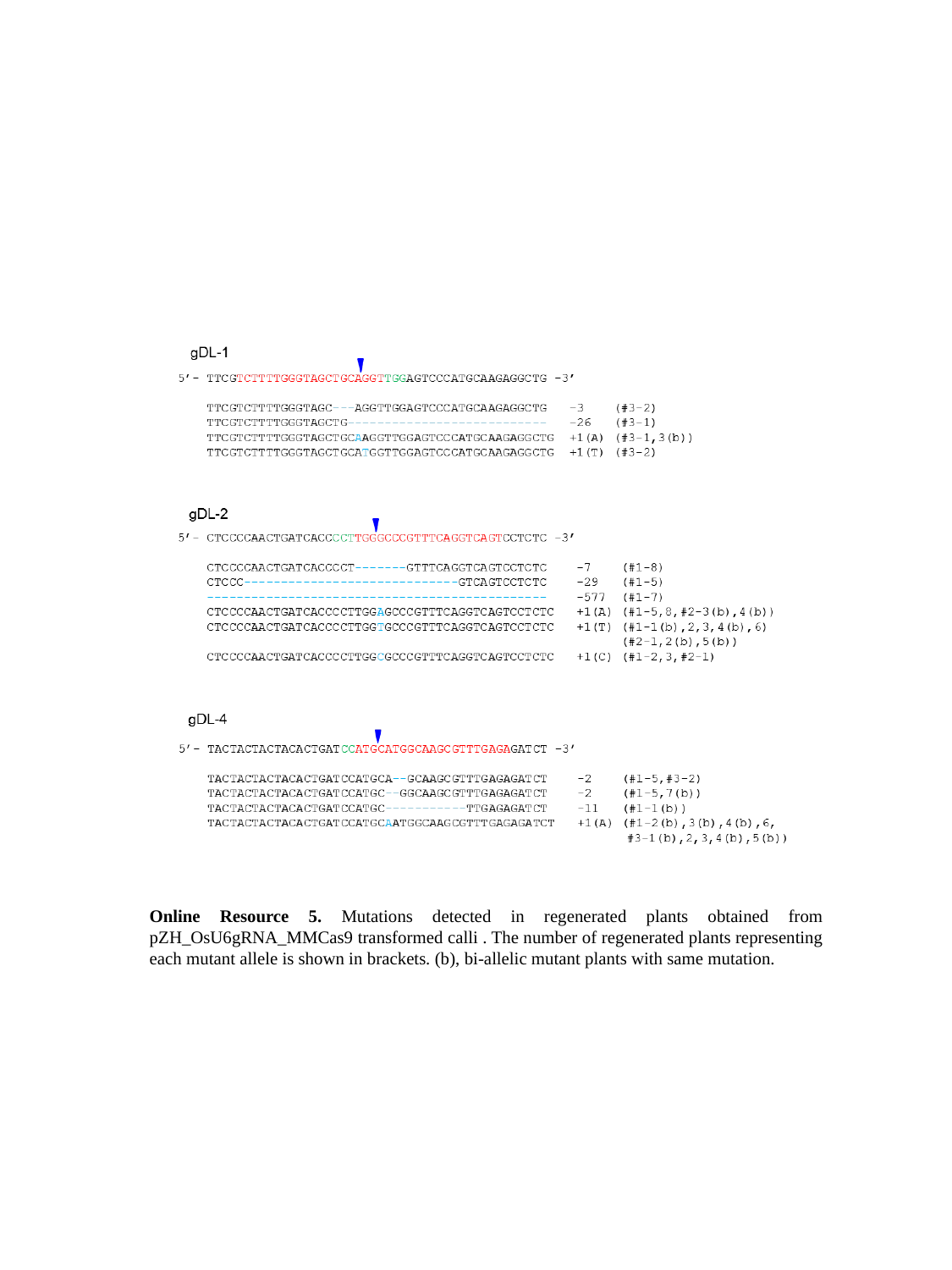

Online Resource 5. Mutations detected in regenerated plants obtained from pZH_OsU6gRNA_MMCas9 transformed calli . The number of regenerated plants representing each mutant allele is shown in brackets. (b), bi-allelic mutant plants with same mutation.

## Slide 7
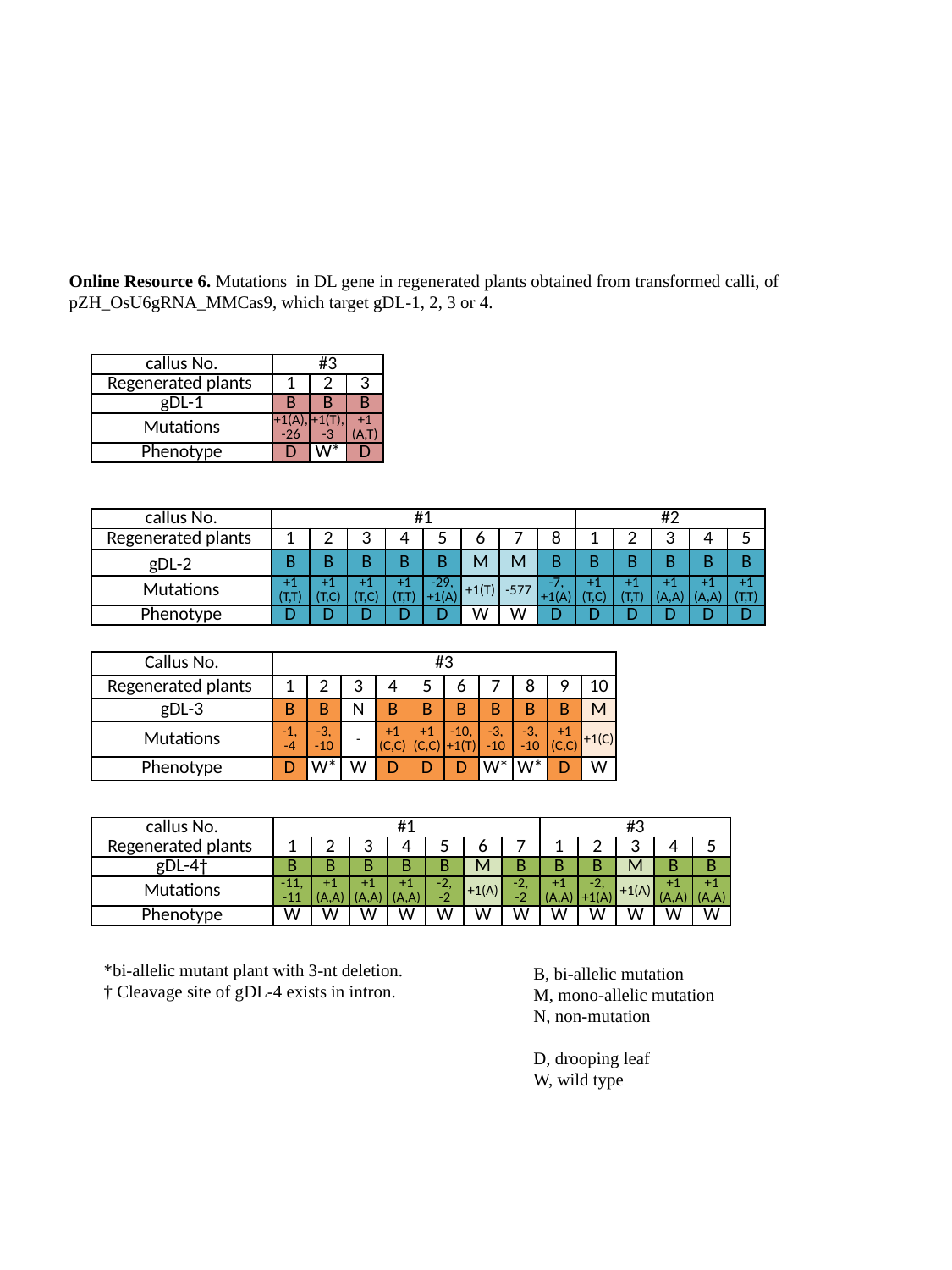

Online Resource 6. Mutations in DL gene in regenerated plants obtained from transformed calli, of pZH_OsU6gRNA_MMCas9, which target gDL-1, 2, 3 or 4.
| callus No. | #3 | | |
| --- | --- | --- | --- |
| Regenerated plants | 1 | 2 | 3 |
| gDL-1 | B | B | B |
| Mutations | +1(A), -26 | +1(T), -3 | +1 (A,T) |
| Phenotype | D | W\* | D |
| callus No. | #1 | | | | | | | | #2 | | | | |
| --- | --- | --- | --- | --- | --- | --- | --- | --- | --- | --- | --- | --- | --- |
| Regenerated plants | 1 | 2 | 3 | 4 | 5 | 6 | 7 | 8 | 1 | 2 | 3 | 4 | 5 |
| gDL-2 | B | B | B | B | B | M | M | B | B | B | B | B | B |
| Mutations | +1 (T,T) | +1 (T,C) | +1 (T,C) | +1 (T,T) | -29, +1(A) | +1(T) | -577 | -7, +1(A) | +1 (T,C) | +1 (T,T) | +1 (A,A) | +1 (A,A) | +1 (T,T) |
| Phenotype | D | D | D | D | D | W | W | D | D | D | D | D | D |
| Callus No. | #3 | | | | | | | | | |
| --- | --- | --- | --- | --- | --- | --- | --- | --- | --- | --- |
| Regenerated plants | 1 | 2 | 3 | 4 | 5 | 6 | 7 | 8 | 9 | 10 |
| gDL-3 | B | B | N | B | B | B | B | B | B | M |
| Mutations | -1, -4 | -3, -10 | - | +1 (C,C) | +1 (C,C) | -10, +1(T) | -3, -10 | -3, -10 | +1 (C,C) | +1(C) |
| Phenotype | D | W\* | W | D | D | D | W\* | W\* | D | W |
| callus No. | #1 | | | | | | | #3 | | | | |
| --- | --- | --- | --- | --- | --- | --- | --- | --- | --- | --- | --- | --- |
| Regenerated plants | 1 | 2 | 3 | 4 | 5 | 6 | 7 | 1 | 2 | 3 | 4 | 5 |
| gDL-4† | B | B | B | B | B | M | B | B | B | M | B | B |
| Mutations | -11, -11 | +1 (A,A) | +1 (A,A) | +1 (A,A) | -2, -2 | +1(A) | -2, -2 | +1 (A,A) | -2, +1(A) | +1(A) | +1 (A,A) | +1 (A,A) |
| Phenotype | W | W | W | W | W | W | W | W | W | W | W | W |
*bi-allelic mutant plant with 3-nt deletion.
† Cleavage site of gDL-4 exists in intron.
B, bi-allelic mutation
M, mono-allelic mutation
N, non-mutation
D, drooping leaf
W, wild type
